# Supplementary material for: Thermophilic bacteria employ a contractile injection system in hot spring microbial mats
Source: ISME J. 2026 Feb 10;20(1):wrag021. doi: 10.1093/ismejo/wrag021 (PMC12998442; doi:10.1093/ismejo/wrag021)
Supplement: Gaisin_et_al_2025_Supplementary_Materials_revision_wrag021 [file gaisin_et_al_2025_supplementary_materials_revision_wrag021.pdf]

Supplementary material

Thermophilic bacteria employ a contractile injection system in hot spring microbial mats

Authors

Vasil A. Gaisin[1,§,*], Corina Hadjicharalambous[1,§], Izabela Mujakić[2], Cristian Villena-Alemany[2], Jiangning Li[1], Michal Koblížek[2], Martin Pilhofer[1,*]

Affiliations

[1] Department of Biology, Institute of Molecular Biology & Biophysics, Eidgenössische Technische Hochschule Zürich, Otto-Stern-Weg 5, 8093 Zürich, Switzerland

[2] Laboratory of Anoxygenic Phototrophs, Institute of Microbiology of the Czech Academy of Sciences, Novohradská 237, 37901 Třeboň, Czechia

[§] Authors contributed equally to this work

* Corresponding authors: pilhofer@biol.ethz.ch, vasil.gaisin@mol.biol.ethz.ch

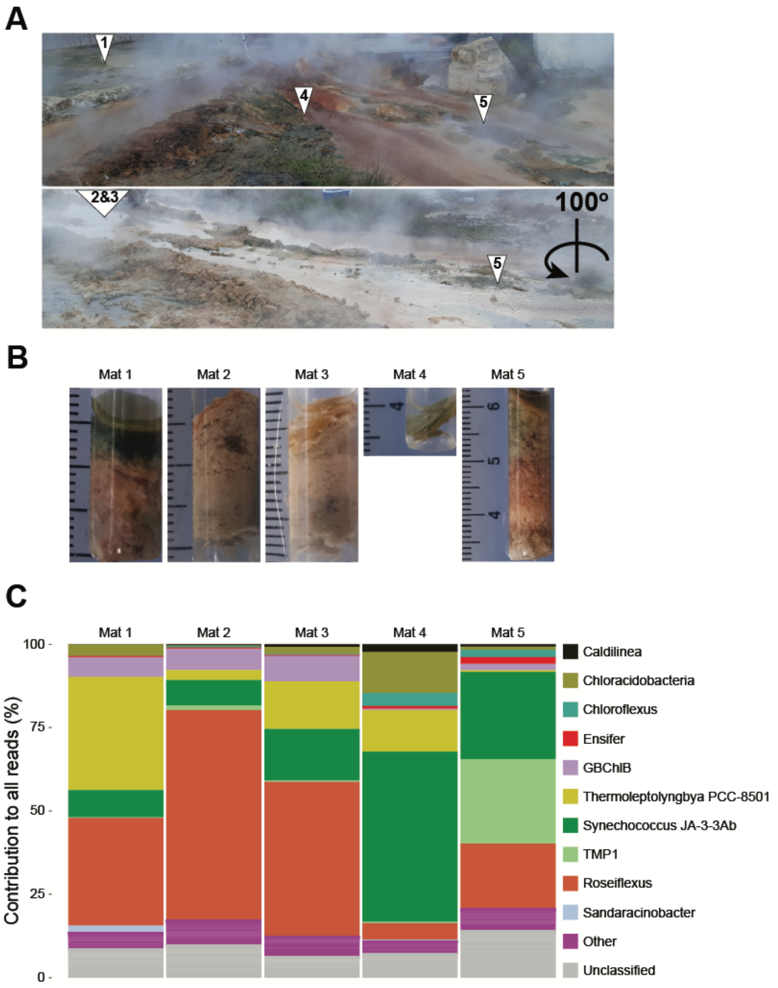

Figure S1. Rupite hot spring accommodates a substantial *Roseiflexus* population. (A) Picture shows the sampling sites at Rupite hot spring from two points of view. (**B**) Pictures show the mat cores collected at the corresponding sampling sites numbered in A. (**C**) Bar plot shows bacterial diversity in the mat samples based on 16S rRNA gene amplicon sequencing results.

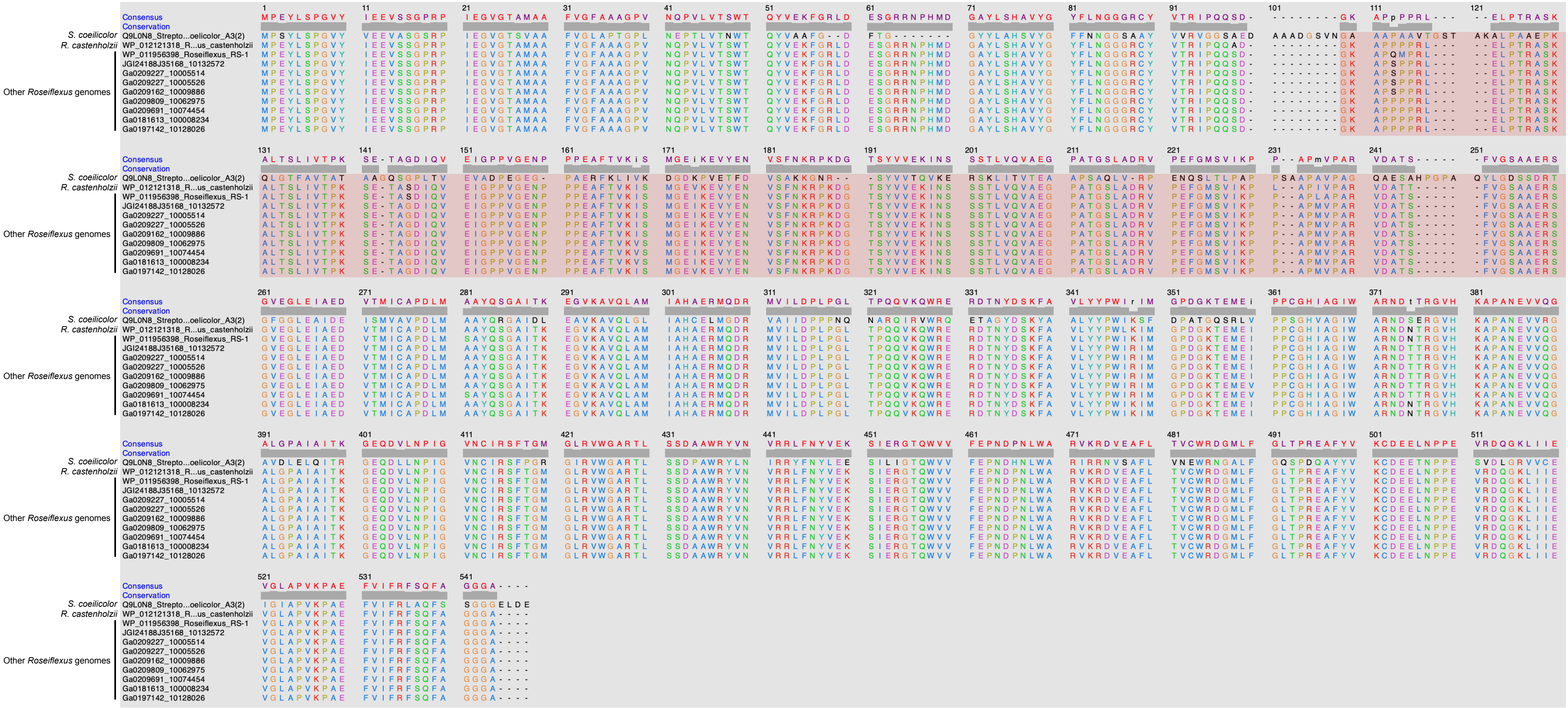

Figure S2. Multiple sequence alignment (MSA) of the sheath protein. The MSA was used to identify a putative domain 3 (highlighted in pink) in the sheath protein retrieved from the *R. castenholzii* genome.

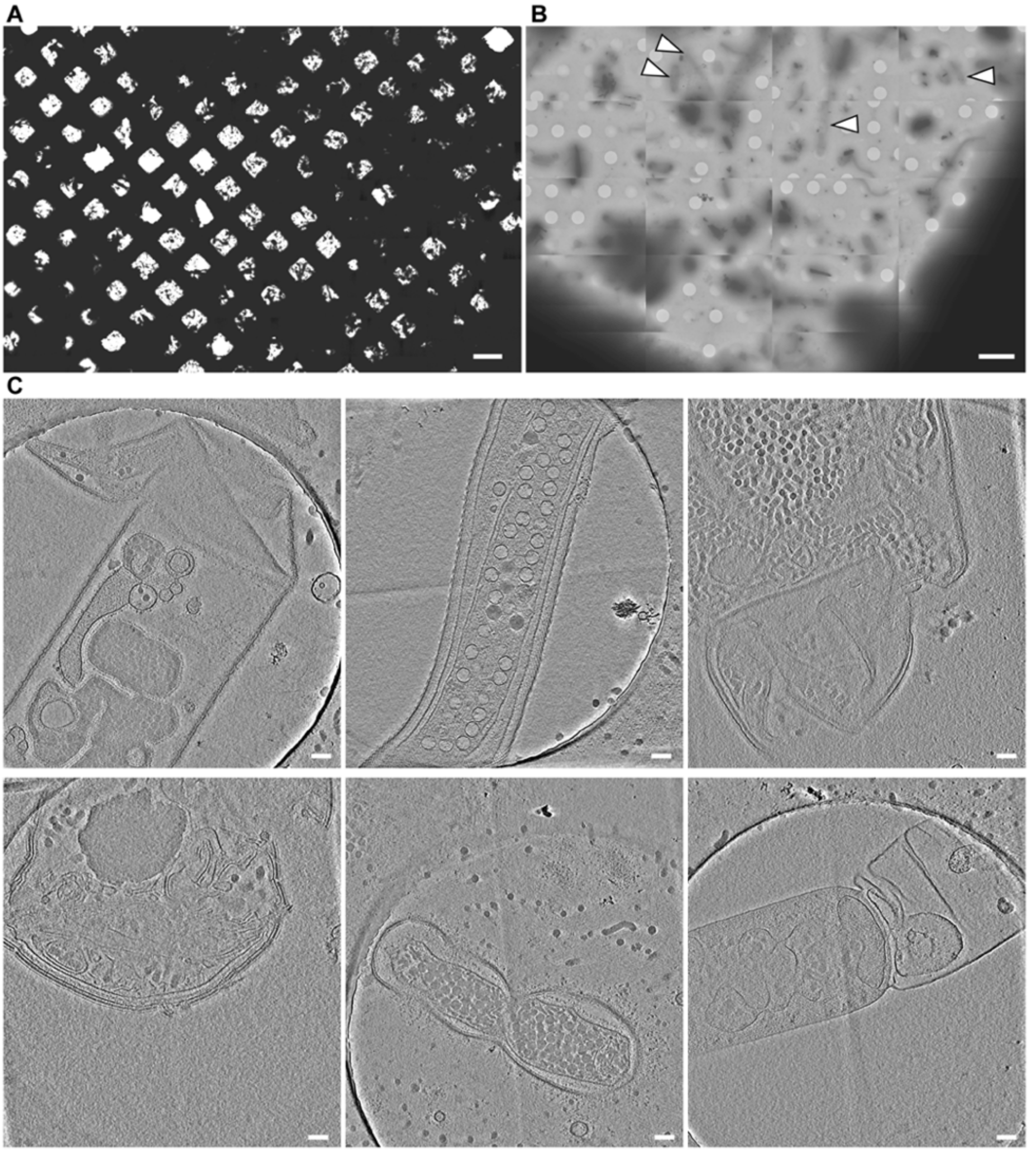

Figure S3. Freeze-thaw cycles damage mat cells. (A) The plunge-frozen mat material is shown in the overview image of an entire EM grid (magnification 135x). (**B**) The damaged cells are seen as “shadow” cells (arrowhead) in an overview image of a grid square (magnification 2,250x). (**C**) Examples of the damaged cells are shown in slices (18 nm thickness) through cryo-tomograms (magnification 19,500x). Scale bars: A, 100 μm; B, 5 μm; C, 100 nm.

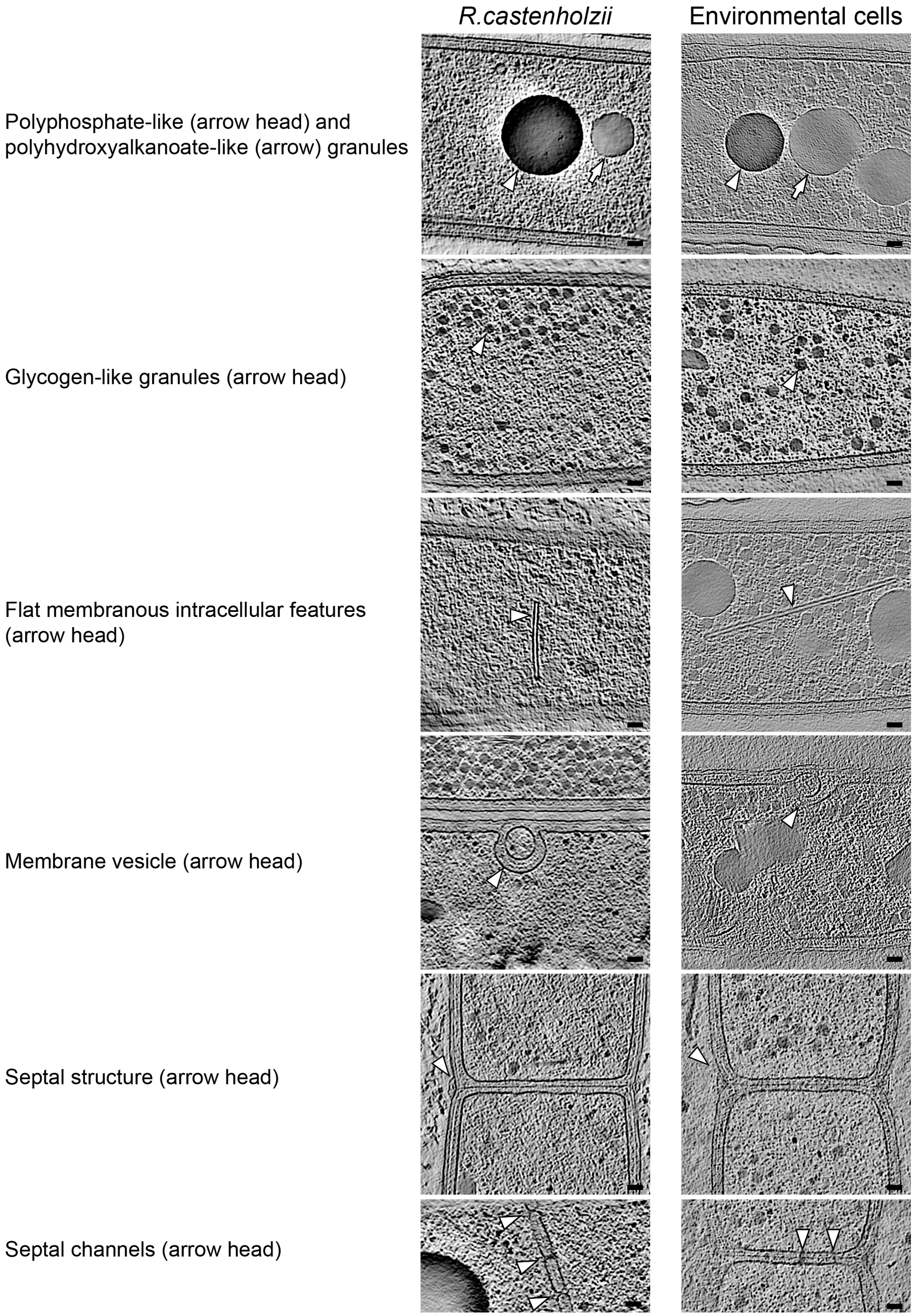

Figure S4. *Roseiflexus* cells in axenic aerobic cultures and mats share unique cellular features. Slices through cryo-tomograms show examples of cellular features in cells of *R. castenholzii* and in environmental *Roseiflexus*-like cells from the mat. Slice thickness: 18 nm. Scale bars: 50 nm.

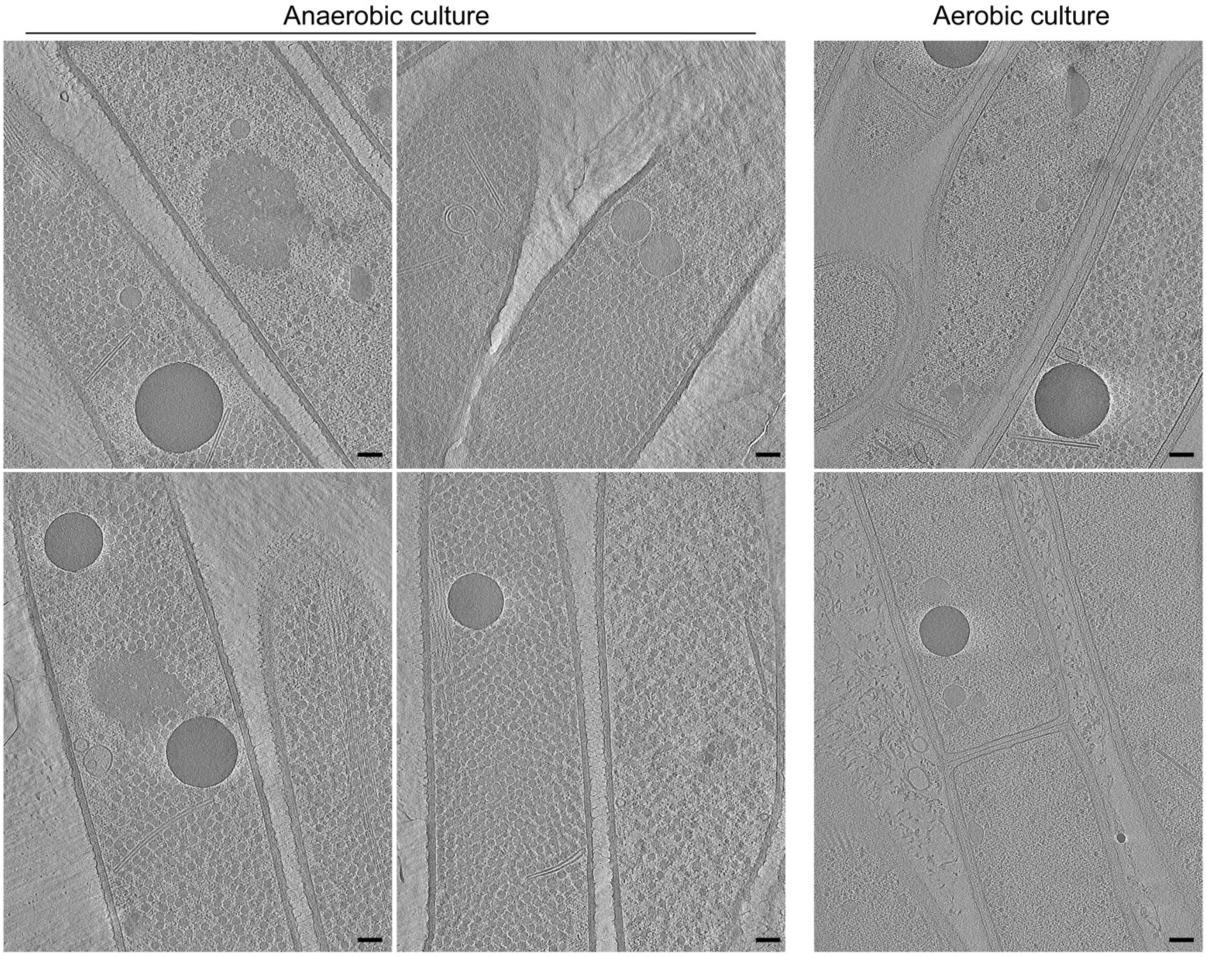

Figure S5. Examples of cryo-tomograms with cells of *R. castenholzii* from the anaerobic and aerobic culture. Slices through cryo-tomograms show examples of *R. castenholzii* cells from anaerobic and aerobic culture. Slice thickness: 18 nm. Scale bars: 100 nm.

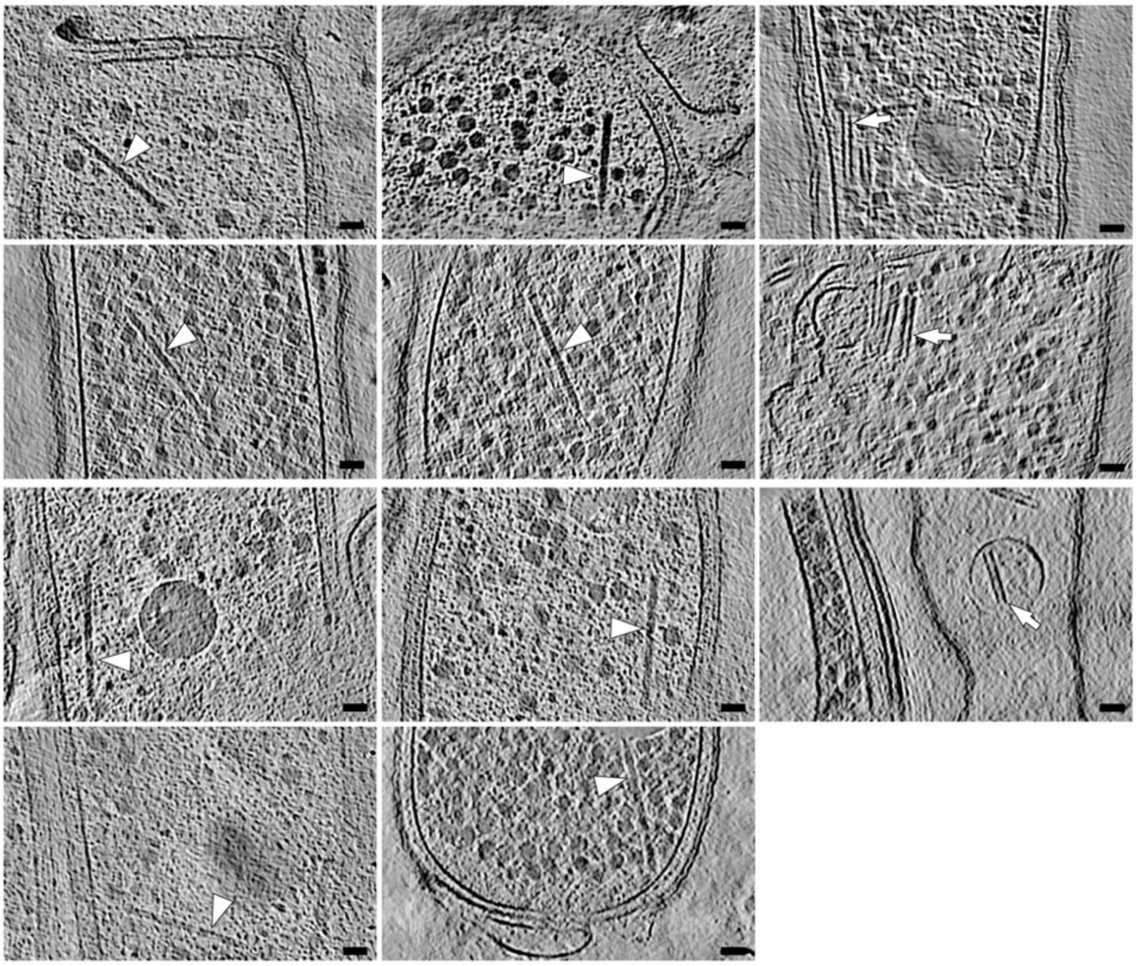

Figure S6. The CIS-like features are seen in mat *Roseiflexus*-like cells.
Slices through cryo-tomograms show examples of CIS-like features observed in environmental *Roseiflexus*-like cells from the mat. Arrowheads indicate extended CIS and arrows indicate contracted CIS. Slice thickness: 13.5 and 18 nm. Scale bars: 50 nm.

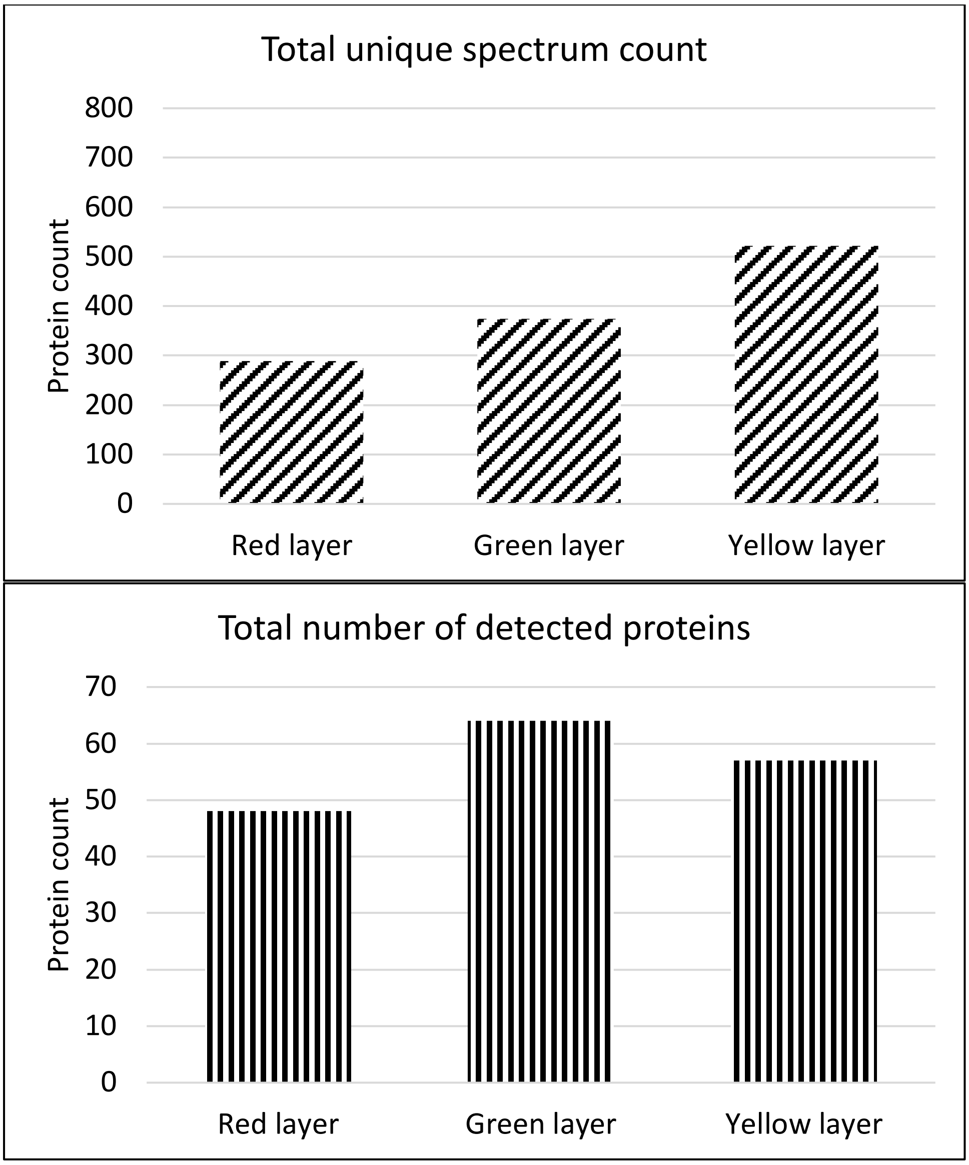

Figure S7. Mass spectrometry with a search against the whole metagenomes (all contigs longer 1 kb from all layers) detected proteins in the CIS purifications of the different mat layers. The top bar graph shows result of the total unique spectrum count, bottom bar graph shows total number of deteced proteins.

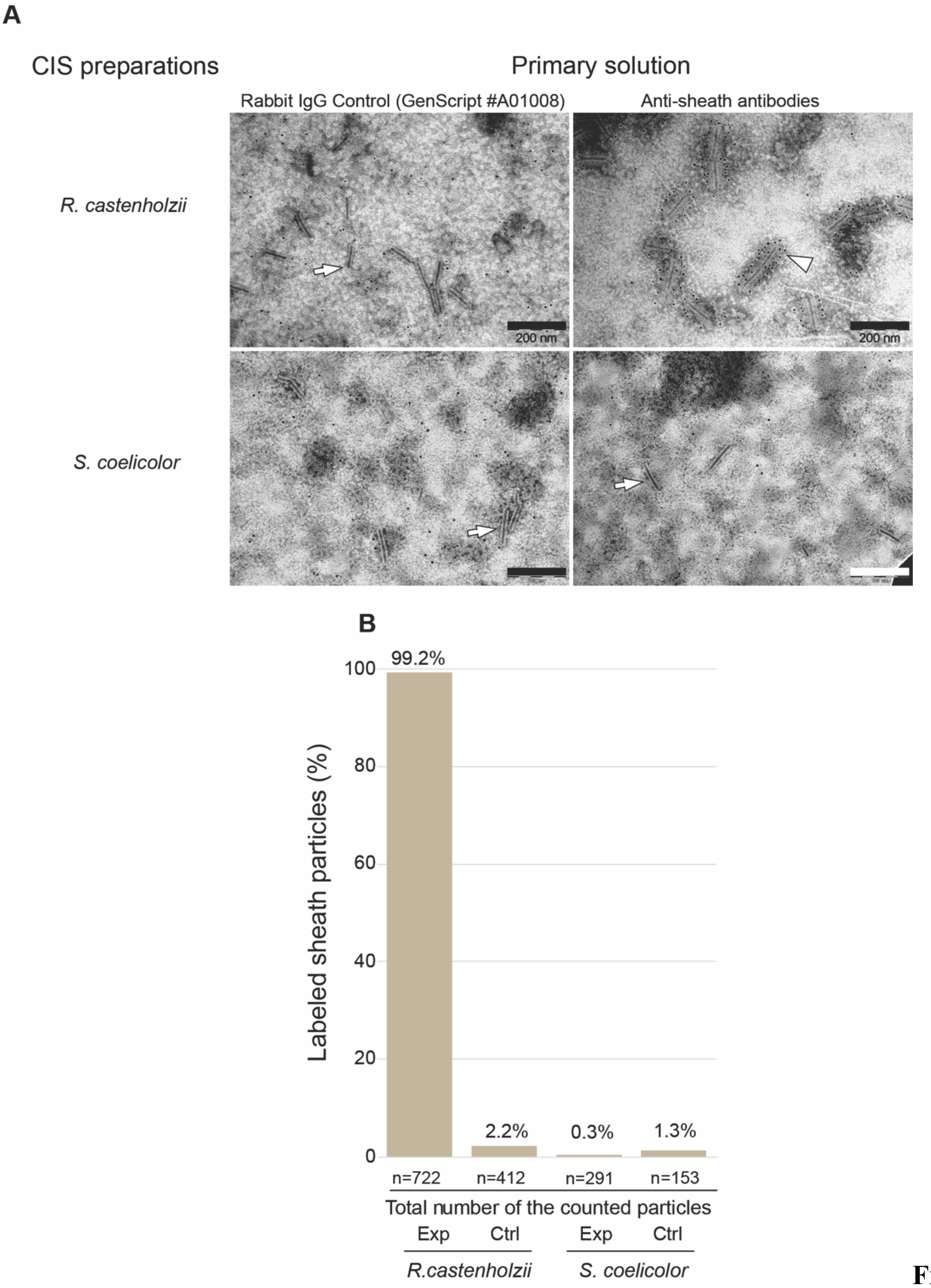

Figure S8. Application of the rabbit anti-sheath antibodies against *Roseiflexus* sheath protein resulted in specific labelling the CIS particles from *Roseiflexus* cells. (**A**) Examples of the original TEM microphotographs show labelled (arrowhead) and unlabelled (arrow) CIS particles from *R. castenholzii* and *S. coelicolor* axenic cultures. Scale bars: 200 nm. (**B**) Bar plot showing number of the labelled CIS particles in the CIS preparations from *R. castenholzii* and *S. coelicolor* axenic cultures. Primary anti-sheath rabbit antibodies were applied for labelling the sheath protein (Exp). Rabbit IgG control was applied as control to check the unspecific binding of anti-rabbit gold-conjugated secondary antibodies (Ctrl). Here, *n* represents the total number of CIS (sheath) particles.

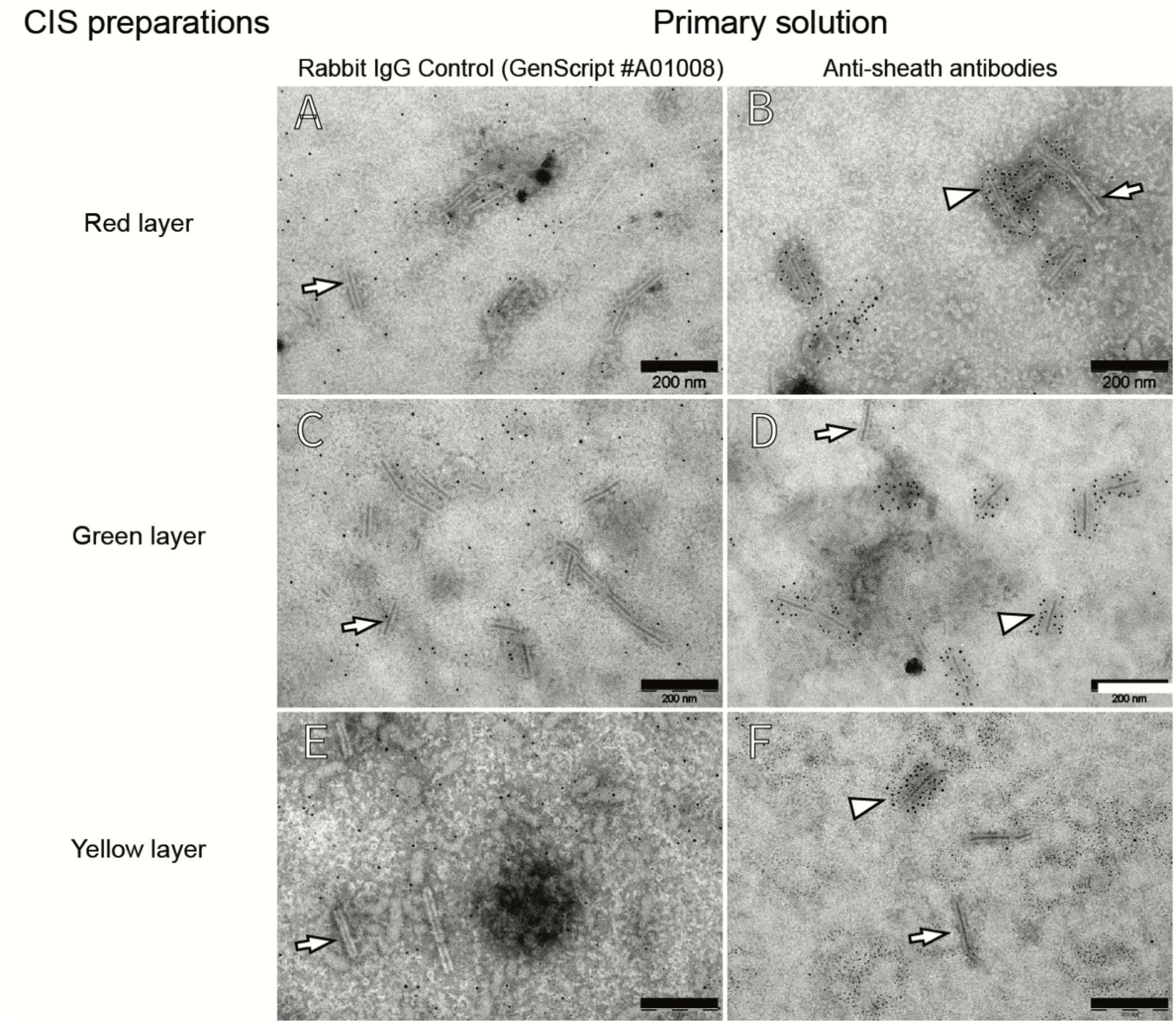

Figure S9. Application of the rabbit anti-sheath antibodies against *Roseiflexus* sheath protein resulted in specific labelling the CIS particles from the mat. Examples of the original TEM microphotographs show labelled (arrowhead) and unlabelled (arrow) CIS particles from the mat layers (Red layer – A and B; Green layer – C and D; Yellow layer – E and F). Scale bars: 200 nm.

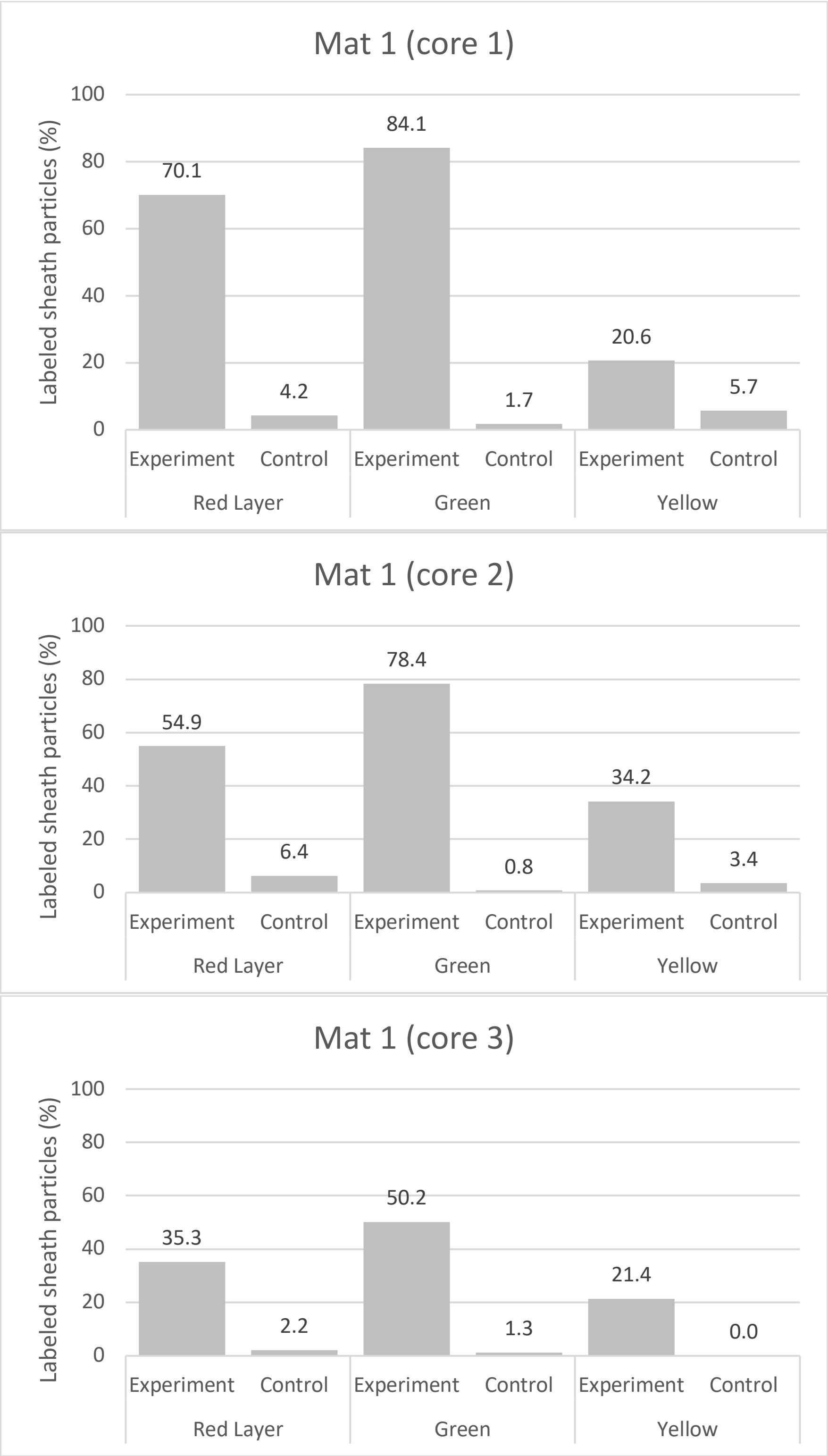

Figure S10. *Roseiflexus* CIS particles represent a major fraction of the CIS particle pool in preparations from the green layer of the mat according to results of the immunogold labelling. Bar plots show the percentage of *Roseiflexus* CIS particles in deferent layers of the three mat cores collected from the mat. *Roseiflexus* CIS particles were counted as immunogold-labelled particles in TEM images (see examples of the images in Figure S8). Number of labelled CIS particles and total number of CIS particles per replicate per layer are presented in Supplementary Table S5.

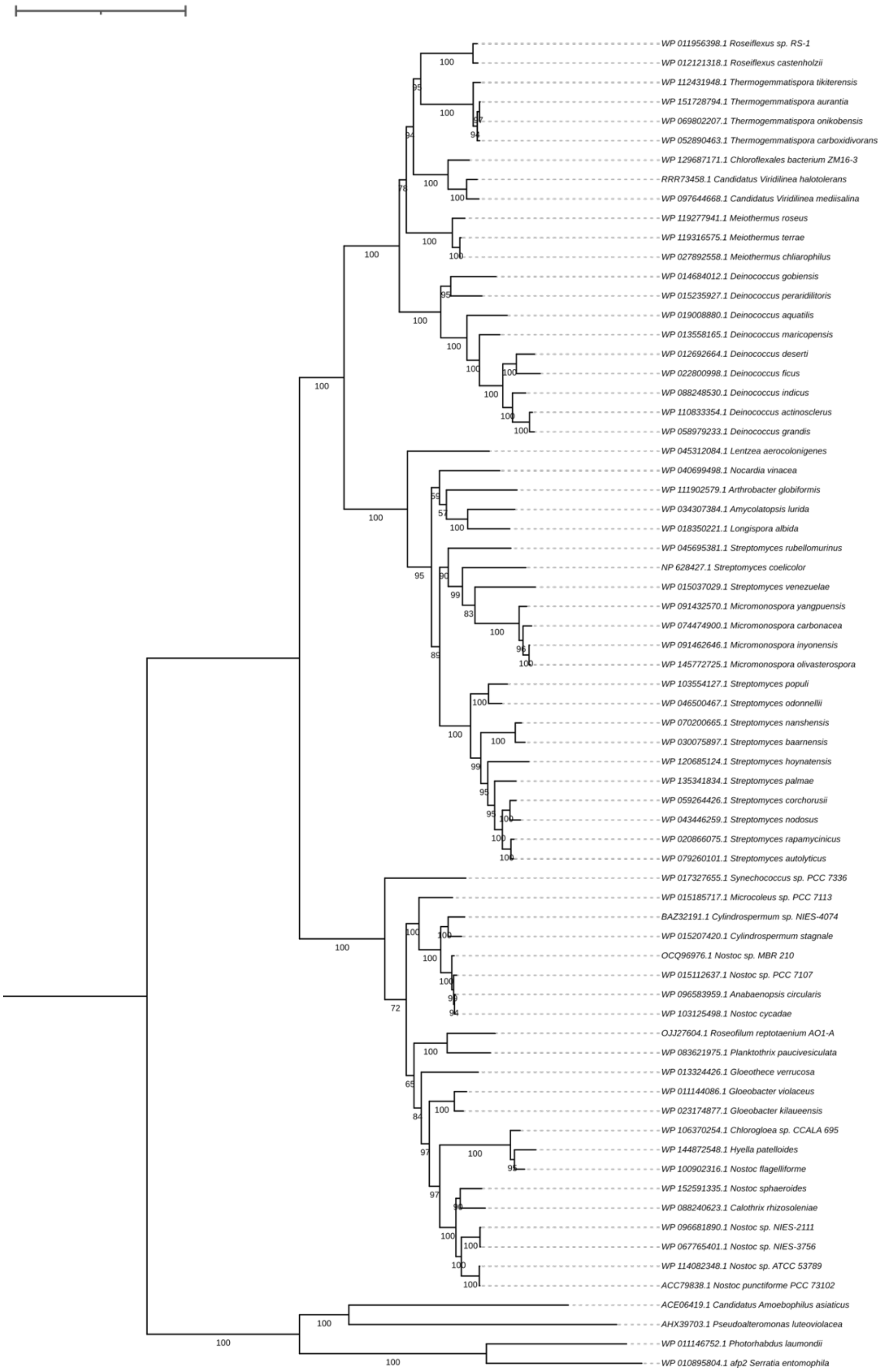

Figure S11. A maximum likelihood phylogenetic tree shows that a clade of the *Roseiflexus*-related CISs clusters with CISs from *Streptomyces* strains within lineage subtype IId. The tree was reconstructed based on analysis of concatenated sheath and baseplate protein sequences using IQ-TREE 1.6.11 with the LG+F+I+G4 model. Scale bar shows number of substitutions per site.

Detailed protocol for protein identification in the CIS preparations

Sample preparation

Proteins were precipitated with trichloroacetic acid (TCA; Sigma-Aldrich) at a final concentration of 5% and washed twice with ice-cold acetone. Protein were taken and reduced with 5 mM TCEP(tris(2-carboxyethyl)phosphine) and alkylated with 15 mM chloroacetamide at 30°C for 30 min. 500 ng of Sequencing Grade Trypsin (Promega) were added for digestion carried out in a microwave instrument (Discover System, CEM) for 30 min at 5 W and 60 °C. Peptides were acidified to perform a stage-tip cleanup using two Empore reversed-phase extraction disks (3M) [1]. The eluted samples were dried to completeness and re-solubilized in 20 µL of MS sample buffer (3% acetonitrile, 0.1% formic acid).

LC-MSMS – Lumos OT/IT DDA

LC-MS/MS analysis was performed on an Orbitrap Fusion Lumos (Thermo Scientific) equipped with a Digital PicoView source (New Objective) and coupled to an M-Class UPLC (Waters). Solvent composition of the two channels was 0.1% formic acid for channel A and 99.9% acetonitrile in 0.1% formic acid for channel B. Column temperature was 50°C. For each sample 200 ng of peptides were loaded on a commercial ACQUITY UPLC M-Class Symmetry C18 Trap Column (100Å, 5 µm, 180 µm x 20 mm, Waters) connected to a ACQUITY UPLC M-Class HSS T3 Column (100Å, 1.8 µm, 75 µm X 250 mm, Waters). The peptides were eluted at a flow rate of 300 nL/min. After a 3 min initial hold at 5% B, a gradient from 5 to 22 % B in 80 min and 22 to 32% B in additional 10 min was applied. The column was cleaned after the run by increasing to 95 % B and holding 95 % B for 10 min prior to re-establishing loading condition.

The mass spectrometer was operated in data-dependent mode (DDA) with a maximum cycle time of 3 s, funnel RF level at 40 %, heated capillary temperature at 275 °C, and Advanced Peak Determination (APD) on. Full-scan MS spectra (300−1’500 m/z) were acquired at a resolution of 120’000 at 200 m/z after accumulation to an automated gain control (AGC) target value of 500’000 or for a maximum injection time of 40 ms. Precursors with an intensity above 5’000 were selected for MS/MS. Ions were isolated using a quadrupole mass filter with 0.8 m/z isolation window and fragmented by higher-energy collisional dissociation (HCD) using a normalized collision energy of 35 %. Fragments were detected in the linear ion trap with the scan rate set to rapid, the automatic gain control set to 10’000 ions, and the maximum injection time set to 50 ms. Charge state screening was enabled, and singly, unassigned charge states and charge states higher than seven were excluded. Precursor masses previously selected for

MS/MS measurement were excluded from further selection for 20 s, applying a mass tolerance of 10 ppm. The samples were acquired using internal lock mass calibration on m/z 371.1012 and 445.1200.

Peptide identification – PEAKS - Scaffold

The acquired MS data were processed for identification using PEAKS Studio XPlus (Bioinformatic Solutions). Spectra were searched against custom proteome database (generated from our metagenomic contigs longer than 1 kb), concatenated to its reversed decoyed fasta database. Methionine oxidation was set as variable modification, Carbamidomethyl (C) as fix modification. Enzyme specificity was set to trypsin allowing a maximum of two missed-cleavages. A fragment ion mass tolerance of 0.02 Da and a parent ion tolerance of 10 ppm were set. Scaffold (Proteome Software Inc., version 5.10) was used to validate MS/MS based peptide and protein identifications. Peptide identifications were accepted if they achieved a false discovery rate (FDR) of less than 0.1% by the Scaffold Local FDR algorithm. Protein identifications were accepted if they achieved an FDR of less than 1.0 % and contained at least 2 identified peptides.

1. Rappsilber J, Mann M, Ishihama Y. Protocol for micro-purification, enrichment, pre-fractionation and storage of peptides for proteomics using StageTips. *Nat Protoc* 2007;**2**:1896–906. doi.org/10.1038/nprot.2007.261
